# Models of Somatic Hypermutation Targeting and Substitution Based on Synonymous Mutations from High-Throughput Immunoglobulin Sequencing Data

**DOI:** 10.3389/fimmu.2013.00358

**Published:** 2013-11-15

**Authors:** Gur Yaari, Jason A. Vander Heiden, Mohamed Uduman, Daniel Gadala-Maria, Namita Gupta, Joel N. H. Stern, Kevin C. O’Connor, David A. Hafler, Uri Laserson, Francois Vigneault, Steven H. Kleinstein

**Affiliations:** ^1^Bioengineering Program, Faculty of Engineering, Bar Ilan University, Ramat Gan, Israel; ^2^Department of Pathology, Yale School of Medicine, New Haven, CT, USA; ^3^Interdepartmental Program in Computational Biology and Bioinformatics, Yale University, New Haven, CT, USA; ^4^Department of Neurology, Yale School of Medicine, New Haven, CT, USA; ^5^Department of Science Education, Hofstra North Shore-LIJ School of Medicine, Hempstead, NY, USA; ^6^Human and Translational Immunology Program, Yale School of Medicine, New Haven, CT, USA; ^7^Department of Immunobiology, Yale School of Medicine, New Haven, CT, USA; ^8^Department of Genetics, Harvard Medical School, Boston, MA, USA; ^9^AbVitro, Inc., Boston, MA, USA

**Keywords:** immunoglobulin, B cell, somatic hypermutation, mutability, substitution, targeting, AID, affinity maturation

## Abstract

Analyses of somatic hypermutation (SHM) patterns in B cell immunoglobulin (Ig) sequences contribute to our basic understanding of adaptive immunity, and have broad applications not only for understanding the immune response to pathogens, but also to determining the role of SHM in autoimmunity and B cell cancers. Although stochastic, SHM displays intrinsic biases that can confound statistical analysis, especially when combined with the particular codon usage and base composition in Ig sequences. Analysis of B cell clonal expansion, diversification, and selection processes thus critically depends on an accurate background model for SHM micro-sequence targeting (i.e., hot/cold-spots) and nucleotide substitution. Existing models are based on small numbers of sequences/mutations, in part because they depend on data from non-coding regions or non-functional sequences to remove the confounding influences of selection. Here, we combine high-throughput Ig sequencing with new computational analysis methods to produce improved models of SHM targeting and substitution that are based only on synonymous mutations, and are thus independent of selection. The resulting “S5F” models are based on 806,860 Synonymous mutations in 5-mer motifs from 1,145,182 Functional sequences and account for dependencies on the adjacent four nucleotides (two bases upstream and downstream of the mutation). The estimated profiles can explain almost half of the variance in observed mutation patterns, and clearly show that both mutation targeting and substitution are significantly influenced by neighboring bases. While mutability and substitution profiles were highly conserved across individuals, the variability across motifs was found to be much larger than previously estimated. The model and method source code are made available at http://clip.med.yale.edu/SHM

## 1 Introduction

During the course of an immune response, B cells that initially bind antigen with low affinity through their immunoglobulin (Ig) receptor are modified through cycles of proliferation, somatic hypermutation (SHM), and affinity-dependent selection to produce high-affinity memory and plasma cells. Current models of SHM recognize activation-induced deaminase (AID), along with several DNA repair pathways, as critical to the mutation process (1). AID initiates SHM by converting cytosines (Cs) to uracils (Us), thus creating U:G mismatches in the Ig V(D)J sequence. If not repaired before cell replication, these mismatches produce C → T (thymine) transition mutations (2). The AID-induced mismatches can alternatively be recognized by UNG or MSH2/MSH6 to initiate base excision or mismatch repair pathways, respectively. These pathways operate in an error-prone manner to introduce the full spectrum of mutations at the initial lesion, as well as spreading mutations to the surrounding bases. Overall, SHM introduces point mutations into the Ig locus at a rate of ∼10^−3^ per base-pair per division (3, 4). While the process of SHM appears to be stochastic, there are clear intrinsic biases, both in the bases that are targeted (5, 6) as well as the substitutions that are introduced (7, 8). Accurate background models for SHM micro-sequence targeting (i.e., hot/cold-spots) and nucleotide substitution would greatly aid the analysis of B cell clonal expansion, diversification, and selection processes. In addition, targeting and substitution models could provide important insights into the relative contributions of the various error-prone DNA repair pathways that mediate SHM.

Computational models and analyses of SHM have separated the process into two independent components (7–11): (1) a targeting model that defines where mutations occur (by specifying the relative rates at which positions in the Ig sequence are mutated), and (2) a nucleotide substitution model that defines the resulting mutation (by specifying the probability of each base mutating to each of the other three possibilities). In experimentally derived Ig sequences, observed mutation patterns are influenced by selection. The affinity maturation process selects for affinity-increasing mutations, while many mutations at structurally important positions in the framework regions are selected against (12). To avoid the confounding influences of selection, most existing models are built using mutation data from intronic regions flanking the V gene (13) and non-productively rearranged Ig genes (6–10, 14). These works have identified several specific motifs as being “hot” or “cold” spots of SHM. Hot-spots include WRCY/RGYW and WA/TW (where W = {A, T}, Y = {C, T} R = {G, A}, and the mutated position is underlined, see for example (5, 6)). Although it has been argued that WRCH/DGYW (where *H* = {A, C, T} and *D* = {A, G, T}) is a better predictor of mutability at C:G bases (15). A single cold-spot motif has also been recognized: SYC/GRS (where *S* = {C, G}) (16). Despite the wide recognition of these specific hot-spot and cold-spot motifs, it is clear that a hierarchy of mutabilities exists that is highly dependent on the surrounding bases (7, 10). More recently, it has been recognized that the profile of nucleotide substitutions may also be dependent on the surrounding bases (8, 17). Modeling SHM targeting and substitution is important for the analysis of mutation patterns since these intrinsic biases can give the appearance of selection due to the particular codon usage and base composition in Ig sequences (17, 18). Moreover, having such a model could shed light on the molecular mechanisms underlying SHM, which are not fully understood.

Previous work has attempted to model the dependencies on surrounding bases, but has been limited to (at most) the targeted base and three surrounding bases (19), mainly due to the relatively small data sets available. The use of intronic regions has also limited the number of motifs that can be modeled (because of the limited diversity of these regions), and non-productively rearranged Ig genes may still be influenced by selection (e.g., if the event rendering the sequence non-productive happened in the course of affinity maturation). In this study, we take advantage of the wealth of data available from high-throughput Ig sequencing technologies to build improved targeting and substitution models for SHM. To avoid the biasing effects of selection, we have developed a new methodology for constructing models from synonymous mutations only, thus avoiding the need to limit analysis to non-productive Ig sequences. The increased data set size allows modeling of dependencies on the surrounding four bases (two bases upstream and downstream of the mutation). These “S5F” (Synonymous, 5-mer, Functional) models confirm the existence of proposed hot- and cold-spots of SHM, but also show much more extreme difference between hot- and cold-spots compared with previous models. We also find that the nucleotide substitution profiles at all bases are dependent on the surrounding nucleotides. The S5F targeting and substitution models can be employed as background distributions for mutation analysis, such as the detection and quantification of affinity-dependent selection in Ig sequences (11, 20). These models improve dramatically the ability to analyze mutation patterns in Ig sequences, and provide insights into the SHM process.

## 2 Results

To develop models for SHM targeting and substitution preferences, we curated a large database of mutations from high-throughput sequencing studies (Table 1). These data were derived from 7 human blood and lymph node samples, and Ig sequencing was carried out using both Roche 454 and Illumina MiSeq next-generation sequencing technologies. In total, the data contained 42,122,509 raw reads, which were processed (see Materials and Methods) to arrive at 1,145,182 “high-fidelity” Ig sequences, which were each supported by a minimum of two independent reads in a sample. These high-fidelity sequences were clustered to identify clones (sequences related by a common ancestor) and one effective sequence was constructed per clone so that each observed mutation corresponded to an independent event. Overall, this process produced a set of 806,860 synonymous mutations that were used to model somatic hypermutation targeting and substitution.

**Table 1:** **Next-generation sequencing data sets used to construct the “S5F” targeting and substitution models**.

Study	Sample	Subject	Tissue	Tech.	Raw reads	Processed reads	Clones	# Mutations (substitution)	# Mutations (targeting)
1	3931LN	1	LN	MiSeq	3,641,633	79,777	16,272	25,307	53,840
1	4014LN	2	LN	MiSeq	3,714,152	106,006	32,972	57,215	106,265
1	4106LN	3	LN	MiSeq	10,917,517	231,387	54,400	108,591	208,338
1	3928LN	4	LN	MiSeq	7,691,509	99,519	76,375	68,051	132,795
2	PGP1-1	5	PBMC	MiSeq	3,851,658	55,606	50,514	23,939	48,558
2	PGP1-2	5	PBMC	MiSeq	3,946,514	59,611	54,374	24,971	50,117
2	PGP1-3	5	PBMC	MiSeq	4,543,353	48,971	45,788	20,865	42,737
2	PGP1-4	5	PBMC	MiSeq	3,121,884	52,844	49,054	23,243	47,049
3	hu420143	6	PBMC	454	178,584	92,055	14,956	23,260	48,838
3	420IV	7	PBMC	454	398,517	248,363	39,047	24,771	50,899
3	PGP1-5	5	PBMC	454	117,188	71,043	12,275	8,209	17,424
Total	–	–	–	–	42,122,509	1,145,182	446,027	408,422	806,860

Tissue types are lymph node (LN) or peripheral blood mononuclear cell (PBMC). The different filters applied to arrive at the number of (synonymous) mutations used for the targeting and substitution models are described in the text. All three studies relate to manuscripts in preparation.

### 2.1 The nucleotide substitution spectrum is affected by adjacent nucleotides

A nucleotide substitution model specifies the probability of each base (A, T, G, or C) mutating to each of the other three possibilities. For example, when a C is mutated, we might find that 50% of the time it is replaced by T, while 30% of the substitutions are to G, and the remaining 20% lead to A. These probabilities may depend on the surrounding bases (i.e., the micro-sequence context), as was previously suggested for mutations at A (17) and more generally (8). To derive a nucleotide substitution model, the set of mutations was filtered to include only those that occurred in positions where none of the possible base substitutions lead to amino acid exchanges. Focusing on positions where only synonymous mutations were possible removes the confounding influence of selection. The resulting 408,422 mutations were analyzed and grouped into “5-mers” according to the germline sequence of the mutated position and surrounding bases (two base-pairs upstream and two base-pairs downstream of the mutated position). For each of the 1024 possible 5-mers (M), a substitution model was derived by calculating $S_{B}^{M}$, the probability that the central base in the 5-mer motif (M) mutates to base B. For example, in the 5-mer CCATC mutations at A are always synonymous whenever this motif starts a reading frame, in which case it codes for a Proline (CCA) followed by a Serine (TCN). In this case, the number of observed mutations that led to each of the other three possible nucleotides (C, G, or T) was recorded: $N_{C}^{CCATC}$, $N_{G}^{CCATC}$, $N_{T}^{CCATC}.$ The maximum likelihood value for the probability that A is substituted by base B is then calculated as:

$$S_{B}^{CCATC}=\frac{N_{B}^{CCATC}}{N_{C}^{CCATC}+N_{G}^{CCATC}+N_{T}^{CCATC}}$$

A bootstrapping procedure was used to estimate 95% confidence intervals (21).

**Figure 1:**
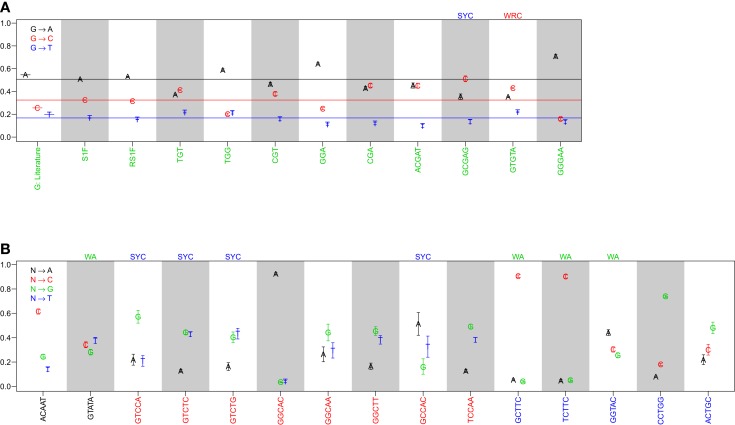
**The substitution profile is significantly influenced by surrounding bases**. Substitution profiles for various micro-sequence contexts are shown for substitutions at **(A)** guanine and **(B)** adenosine, cytidine, and thymidine. G: literature indicates values estimated by Smith et al. (7), while S1F and RS1F refer to models estimated using the methods proposed here using all (replacement and silent) or only silent mutations, respectively, and averaging over surrounding bases. 3-mer motifs were estimated using silent mutations and dependencies on the immediately adjacent bases (S3F), while 5-mer motifs refer to the complete S5F model. Horizontal lines in **(A)** indicate the substitution values for the S1F model following the color scheme shown in the legend. Motifs that fall into one of the standard hot or cold-spots categories are indicated by the motif above the column.

Comparison of the substitution profiles for different 5-mer motifs with the same central base clearly showed the significant influence of surrounding bases. As an example, Figure 1A shows how the profile of substitutions at G changes for several different 5-mers (ACGAT, GCGAG, GTGTA, and GGGAA). Such dependencies were identified for every base (A, T, G, and C) (Figure 1B and Figure S1 in Supplementary Material). The importance of including two bases upstream and downstream was confirmed by comparing these profiles with analogous profiles that only account for the immediately adjacent bases (3-mer motifs) (Figure 1A). For the 3-mer CGA, G → C and G → A substitutions were equally likely (45% and 43% of substitutions, respectively), while G → C substitutions were significantly more likely than G → A in the context of the GCGAG motif (51% and 35% of substitutions, respectively). If one ignores neighboring nucleotides, the substitution profiles were qualitatively similar to previous estimates (7), although significant quantitative differences were apparent (presumably due to the much larger size of the dataset compiled here). Thus, nucleotide substitution profiles at every base are significantly affected by adjacent nucleotides, including at least two bases on either side of the mutating base.

#### 2.1.1 The complete substitution model for somatic hypermutation is not strand-symmetric

It is not possible to estimate substitution profiles for all 5-mer motifs using the above methodology because: (1) not all 5-mers appear within the set of Ig sequences, and (2) some 5-mers (such as NANNN) can never appear in a context where all substitutions at the central (underlined) base are synonymous. Among the 11 datasets used here, these issues prevent estimation of the substitution profiles for 717 of the 1024 5-mers. For the profiles that could be directly estimated, there was a high correlation (on average Pearson R = 0.63) between different individuals (Figures S2 and S3 in Supplementary Material), and so all the samples were combined to estimate a single substitution model. To infer values for the missing motifs, four methods were evaluated. In the first method (“inner 3-mer”), the substitution profile for each missing 5-mer was inferred by averaging over profiles for all 5-mers with the same 3-mer core (i.e., for which the middle three bases were shared). In the second and third methods, missing values were replaced by averaging over motifs sharing the two bases upstream and downstream of the mutated base, respectively. In the fourth method (“hot-spot”), the missing substitution profile was inferred by averaging over 5-mers sharing the two upstream bases when the mutated position was “C” or “A,” and two downstream bases when the mutated position was “G” or “T.” This final option was motivated by the dependencies of known “hot” and “cold” spots for SHM targeting (5, 6). To choose between these four methods, we compared their performance on 5-mers that could be directly estimated from the data. Specifically, we calculated the correlation between the inferred and directly estimated ratios for the parameter *R*, which was defined as the ratio between the highest substitution probability with the next highest one for a given 5-mer (Table 2). Pearson and Spearman coefficients were both used in order to be robust to the linear dependency assumption, and they yielded comparable results. While the “hot-spot” method clearly had the worst performance, the other three methods resulted in very similar models. The “inner 3-mer” method produced the highest Pearson correlation (0.4, see Table 2) and was chosen as the basis to infer missing values. We refer to the resulting substitution model as a “S5F” model since it is based on Synonymous mutations at 5-mers in Functional Ig sequences. In contrast to previous studies (8), there was no significant correlation between substitution values of 5-mers and their reverse complements (Pearson correlation of 0.005, Spearman correlation of 0.087), suggesting that at least one component of the substitution mechanism is not strand-symmetric.

**Table 2:** **Correlation coefficients for inferring missing mutability/substitution values**.

Model	Correlation	Middle	Upstream	Downstream	Hot spots
Substitution	Pearson	0.40	0.37	0.15	0.04
	Spearman	0.20	0.24	0.23	0.09
Mutability	Pearson	0.58	0.57	0.61	0.73
	Spearman	0.61	0.58	0.64	0.79

### 2.2 The hierarchy of motif mutabilities is conserved across individuals

The mutability of a motif is defined here as the (non-normalized) probability of the central base in the motif being targeted for SHM relative to all other motifs. Similar to the substitution model, the targeting model was based on 5-mer motifs, including the two nucleotides immediately upstream and downstream of the mutated base. The use of a 5-mer model is motivated by the well-known WRCY hot-spot (where the underlined C is targeted for mutation), and its reverse-complement (RGYW) which, when taken together, create dependencies with the two bases on either side of the mutating base.

When estimating the mutability (μ) for a motif (M), it is critical to account for the background frequency of M. To see why this is the case, consider the extreme example of a sequence composed of all C nucleotides. Since all mutations will occur at CCCCC motifs, one might consider this motif a hot-spot, except that its background frequency is 100% so it is actually targeted at the expected frequency. When calculating mutabilities it is also important to avoid statistical artifacts due to heterogeneity (e.g., the Simpson paradox (22)). Thus, Ig sequences were first analyzed individually since each has a different background 5-mer distribution. These individual-sequence targeting models were then combined into a single aggregated targeting model for each data set. Estimating the relative mutabilities of 5-mer motifs for an individual Ig sequence involves two steps: (1) Calculating the background frequency of the different 5-mers based on the germline (unmutated) version of the sequence, and (2) creating a table of the 5-mers that were mutated in the sequence. To avoid the confounding influence of selection, only mutations that were synonymous (i.e., that do not produce an amino acid exchange in the germline context) were included in the analysis. Note that these criteria are slightly different from those used in the substitution model. In the substitution model, mutations were used only where all possible mutations at that position had to be synonymous, while all synonymous mutations were considered for mutabilities (see Table 1).

For each of the 1024 possible 5-mers motifs (M) in each Ig sequence, the background frequency (*B*_M_) was calculated as follows:

(1) $$B_{M}=\sum_{i}\sum_{b}\;S_{b}^{M}I_{\vec{GL}}\left(i,M,b\right)$$

where *i* is summed over all (non-N) positions in the Ig sequence, M is the 5-mer nucleotide sequence centered at position *i* and *b* includes all possible nucleotides ({A, C, T, G}). In this equation *GL* is a vector containing the nucleic content of each position in the germline sequence, $S_{b}^{M}$ is the relative rate at which the center nucleotide in M (*GL*[*i*]) mutates to *b* (as estimated in the previous section, and where $S_{GL\left[i\right]}^{M}=0$) and $I_{\vec{GL}}$(*i*, *M*, *b*) is an indicator function that is 1 in cases where the 5-mer surrounding *GL*[*i*] is M and a mutation in position *i* from *GL*[*i*] to *b* results in a synonymous mutation (and 0 otherwise). A similar array was also calculated for the mutated positions:

(2) $$C_{M}=\sum_{i}\;I_{\vec{GL},\vec{OS}}\left(i,M\right)$$

where *i* is summed over all (non-N) positions in the observed Ig sequence (OS), and the indicator function $I_{\vec{GL},\vec{OS}}$ (i, M) is 1 in cases where the 5-mer surrounding *GL*[*i*] is M and a mutation in position *i* from *GL*[*i*] to *OS*[*i*] is synonymous and 0 otherwise. After calculating the arrays $\vec{C}$ and $\vec{B}$, a mutability score, μ, was defined for each motif M in the vector (for sequence *j*) as:

(3) $$\mu_{M}^{j}=C_{M}^{j}∕B_{M}^{j}$$

which was then normalized to one:

(4) $${\bar{\mu }}_{M}^{j}=\mu_{M}^{j}∕\sum_{\text{m}}\;\mu_{m}^{j}$$

where m is an index spanning all positions in ${\vec{\mu }}^{j}$. Note that $\mu_{M}^{j}$ is not defined wherever $B_{M}^{j}=0$ (i.e., the motif M does not appear in the Ig sequence, or can not admit any synonymous mutations). Finally, a single mutability score is generated for each 5-mer motif (M) as the weighted average of the mutabilities scores for each sequence *j* (${\bar{\mu }}_{M}^{j}$), where weights correspond to the number of synonymous mutations in the sequence $\left(\sum_{M}\;C_{M}^{j}\right)$. This process resulted in an array of (relative) mutabilities, *μ*_M_ for each of the 5-mers observed in the dataset. The resulting vector was renormalized so that the mean mutability was one.

#### 2.2.1 Inference of missing values to complete targeting model

It was not possible to estimate mutabilities for 468 of the 1024 possible 5-mer motifs because not all 5-mers appeared within the set of Ig sequences. The same four methods tested for inferring missing values in the substitution model were also tested to infer these mutabilities (see 2.1.1 and Table 2). The “inner 3-mer” method produced a Pearson correlation of 0.58 (0.61 for Spearman), while the “hot-spot” method had a correlation of 0.73 (0.79 for Spearman). Thus, in contrast to the nucleotide substitution model, mutabilities were best predicted by averaging over 5-mers which shared the two upstream bases when the mutated position was “C” or “A,” and two downstream bases when the mutated position was “G” or “T.” This result is consistent with the expected influence of the classic SHM hot-spot (WRCY/RGYW).

#### 2.2.2 Targeting is conserved across individuals

**Figure 2:**
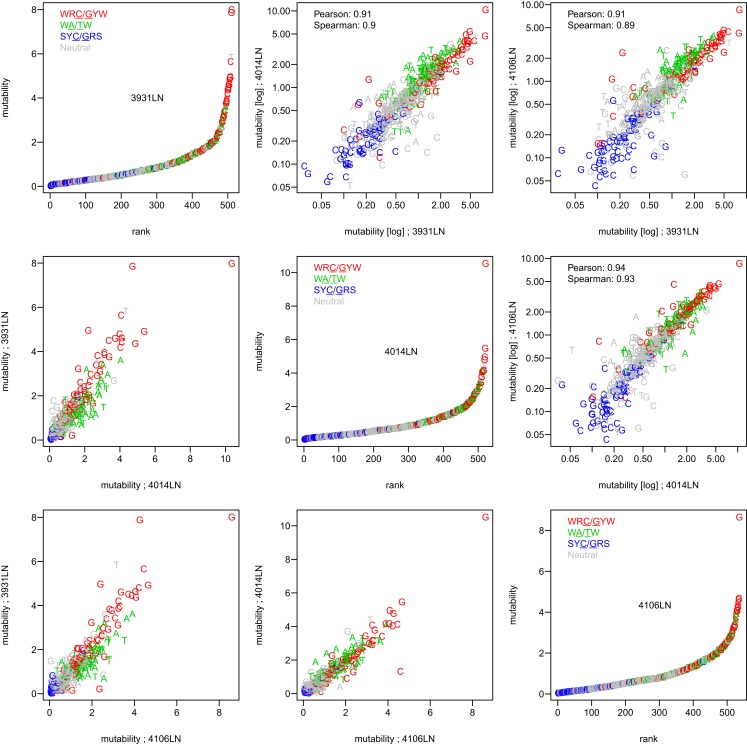
**The S5F targeting model is consistent across individuals**. Targeting models were constructed independently for each of the samples listed in Table 1. Estimated values for all 5-mer motifs derived using lymph node samples from three individuals (3931LN, 4014LN, and 4106LN) are shown along the diagonal. Mutability values are ranked (from lowest to highest) and color coded by their category (WRC/GYW are red, SYC/GRS are blue, WA/TW are green, and the rest are gray). Symbols indicate the mutated nucleotide (in the center of the 5-mer). Correlations between the mutabilities for all 5-mer motifs across individuals are shown in the upper (log-log scale) and lower (linear scale) triangles.

**Figure 3:**
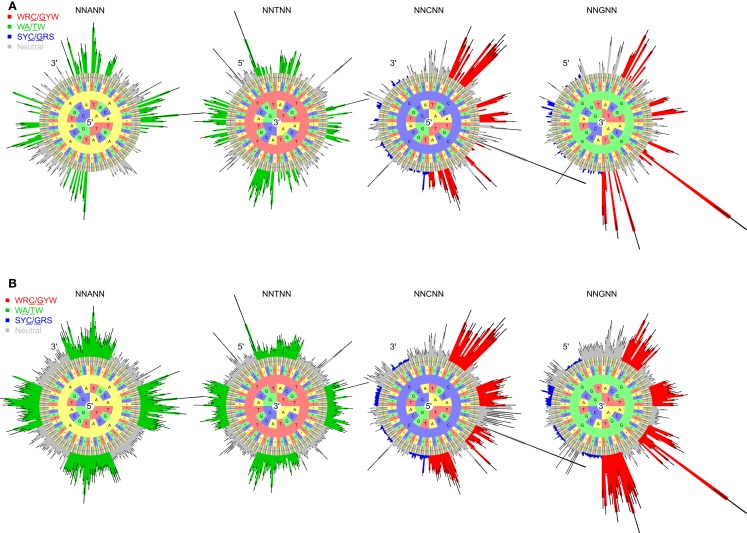
**“Hedgehog” plots for the S5F targeting model for somatic hypermutation targeting A, T, C, and G nucleotides (individual circles)**. **(A)** 5-mer mutabilities estimated directly from the Ig sequencing data. **(B)** The complete S5F targeting model after inferring values for missing 5-mer motifs. Bars radiating from each circle depict the mutability as a function of surrounding bases. The inner-most ring in the circle corresponds to two positions upstream (5′) of the mutated base for A and C, and two positions downstream (3′) of the mutated base for T and G. Bar colors indicate known hot/cold-spot motifs (WRC/GYW are red, WA/TW are green, SYC/GRS are blue, and “neutral” are gray). Each plot corresponds to a different mutated nucleotide. Error bars indicate confidence intervals based on the middle 50% quantiles of the mutability value distribution across the set of samples.

To test whether the micro-sequence specificity of SHM was conserved across individuals, separate targeting models were constructed for each of the 11 samples in our study (Table 1). Comparison of the motif mutabilities between pairs of samples showed that the models were highly consistent, with Pearson correlation ∼0.9 (Figure 2 and Figure S4 in Supplementary Material). Thus, we combined the data from all of the samples and generated a single targeting model, with confidence intervals based on the middle 50% quantiles of the mutability across samples. As with the substitution model, we refer to this targeting model as a “S5F” model. In order to visualize this model, we created “hedgehog” plots to display the directly estimated mutability values and the complete S5F model (Figures 3A,B, respectively).

#### 2.2.3 The true “hotness” of SHM hot-spots

**Figure 4:**
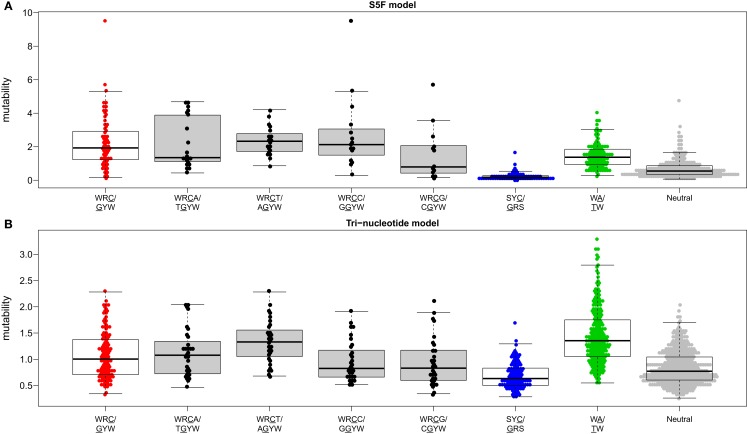
**The hierarchy of hot/cold-spot motifs**. Mutability values predicted by the **(A)** S5F model or **(B)** the tri-nucleotide model of Shapiro et al. were grouped by the type of motif. Boxes borders correspond to the first and fourth quartiles while the horizontal bar inside the box corresponds to the median of the distribution. Hot/cold spot motif groups are plotted using the same colors as in Figure 3B with open boxes. WRCN/NGYW motifs are plotted using filled boxes.

**Figure 5:**
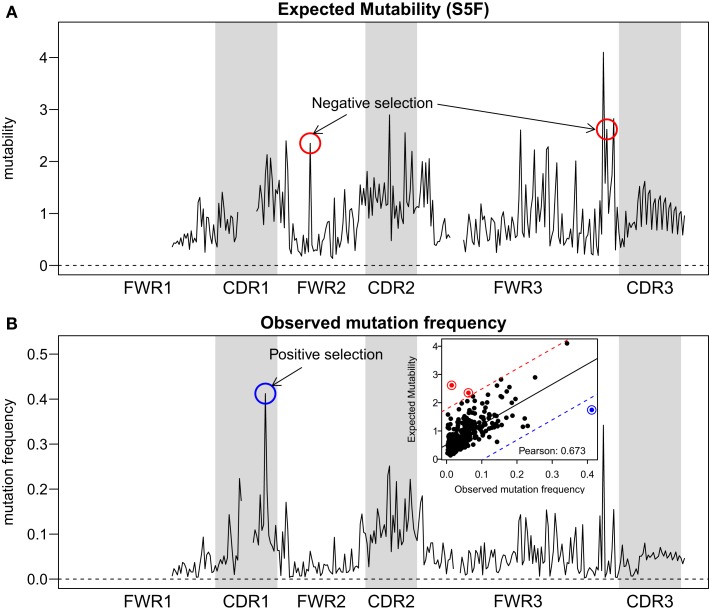
**Comparison between expected and observed somatic hypermutation targeting**. **(A)** The predicted mutability from the S5F model and **(B)** the observed mutation frequency from sample 3931LN (averaged over all clones) for each position in the Ig sequence (IMGT-aligned along the *x*-axis). The correlation across positions (points) is shown in the inset of **(B)**. Two positions with evidence of negative selection (red circles) and one position with positive selection (blue circles) are indicated. The threshold for calling a position with significant selection was set to 3 SD away from the linear regression line (shown as a solid line in the inset, with thresholds plotted as dashed lines).

**Figure 6:**
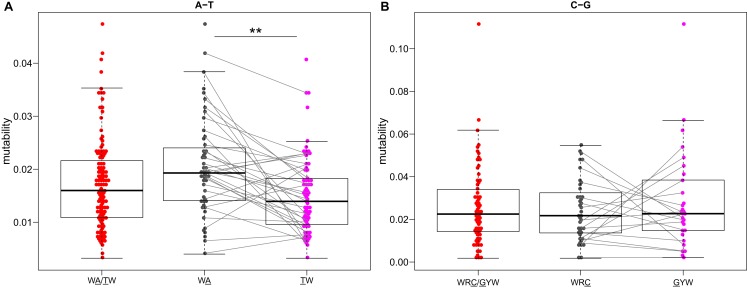
**Somatic hypermutation targeting at C/G, but not A/T, hot-spots is strand symmetric**. Mutability values directly estimated by the S5F model (Figure 3) for **(A)** WA and **(B)** WRC “hot-spot” motifs are compared between different strands. Lines connect reverse-complement motifs for cases where both could be directly estimated from the data. **P < 5 × 10^−4^ by a paired Mann–Whitney–Wilcoxon test.

Visual inspection of the “hedgehog” plots (Figure 3B) shows clearly that the S5F model is consistent with known micro-sequence preferences for SHM (5, 6). WRC/GYW and WA/TW hot-spot motifs are generally more mutable, while SYC/GRS cold-spot motifs generally show the lowest mutability. However, the mutability of “hot-spot” motifs was observed to be highly variable. There is a 62.7-fold difference between the most mutable (GGGCA, mutability = 9.56) and least mutable (TGCGA, mutability = 0.15) WRC/GYW hot-spot motif. Indeed, ∼10% of so-called “hot-spots” had mutabilities that were lower than the mean mutability for “neutral” motifs (Figure 4A). This high variance was especially obvious when looking at the subset of WRCA/TGYW hot-spot motifs, and may help explain why WRCH/DGYW has been proposed to be a better predictor of mutation at C:G compared with WRCY/RGYW (15). The mutabilities estimated by the S5F approach paint a qualitatively different picture of SHM when compared with those estimated by the existing tri-nucleotide model of Shapiro et al. (10). In the S5F model, the average mutability of motifs that correspond to the WRC/GYW SHM hot-spot was 3.2-fold higher than neutral motifs, and 9.6-fold higher than the mutability of motifs corresponding to the cold-spot SYC/GRS (Figure 4A). Using the tri-nucleotide model, hot-spots were only 1.3-fold and 1.6-fold more mutable than neutral and cold-spots, respectively (Figure 4B). In addition, in direct opposition to the S5F model, the tri-nucleotide method predicted that A/T hot-spots (WA/TW) were more mutable than C/G hot-spots (WRC/GYW). The mutabilities estimated by the S5F model better predicted the positional-distribution of *in vivo* mutations. The Pearson correlation between the expected mutability and observed mutation frequency calculated over IMGT-numbered positions in 12,000 sequences derived from a variety of germline segments was 0.67 and 0.47 for the S5F and tri-nucleotide models, respectively (Figure 5 and Figure S5 in Supplementary Material). In both methods, deviations from the expected frequencies that likely reflect both positive and negative selection were observed (Figure 5). The observation of position-specific signals suggests that there is something generic about the Ig structure at these positions, and may help refine traditional definitions of the complementarity determining regions (CDR) and framework regions (FWR) (see also (23)). Consistent with previous studies (24), the S5F model displayed significant strand-bias at A/T hot-spots, but not C/G hot-spots (Figure 6). Overall, the S5F targeting model provides a new view of SHM with hot-spots being significantly more targeted (and significantly more variable) than previously thought.

## 3 Materials and Methods

### 3.1 High-throughput Ig sequencing data sets

A total of 11 human Ig repertoires were sequenced from blood and lymph node samples from 7 different individuals. Next-generation sequencing was carried out using Illumina MiSeq 250 base-pair paired-end reads (8 samples) and Roche/454 GS FLX (3 samples). Details are provided in Table 1. These samples were originally collected and sequenced as part of three ongoing studies (manuscripts in preparation).

#### 3.1.1 Illumina MiSeq data

Human lymph node specimens were collected under an exempt protocol approved by the Human Research Protection Program at Yale School of Medicine. Tissues were processed and RNA isolated as previously described (25). Blood samples were collected under the approval of the Personal Genome Project (26). Total RNA was immediately extracted from each blood sample and stored at −80°C until use. To carry out sequencing, mRNA was reverse transcribed into cDNA using gene-specific primers mapping to the constant region of the Ig heavy chain. Resultant cDNA was tagged with 17 nucleotide single-molecule barcodes and amplified by PCR in a multiplex reaction using primer sets for all possible V-regions (*n* = 45) and isotype/J-regions (*n* = 6) to generate heavy chain transcripts. The amplified library was tagged with barcodes for sample multiplexing, PCR enriched, and annealed to the required Illumina clustering adapters. High-throughput 250 base-pair paired-end sequencing was performed using the Illumina MiSeq platform. Raw reads were exported without the sample barcodes and Illumina clustering adapters.

#### 3.1.2 Roche/454 GS FLX data

Blood samples were collected under the approval of the Personal Genome Project (26). Total RNA was immediately extracted from each blood sample and stored at −80°C until use. Ig heavy chain mRNA were reverse-transcribed using a pool of 6 primers specific to the Ig constant regions and cDNA was amplified using 16 cycles of PCR with a pool of 46 V-region-specific primers and 6 nested constant region primers. Following ligation of 454-compatible sequencing adapters, the expected heavy chain V gene fragments were purified using PAGE. Each sample was uniquely barcoded during the ligation process, allowing subsequent mixing of all the samples into one common reaction sample (performed independently for each replicate run). Emulsion PCR and 454 GS FLX sequencing were performed directly at the 454 Life Sciences facility according to the manufacturer’s standard protocols.

### 3.2 Sequencing data pre-processing

Raw sequencing reads were filtered in several steps to identify and remove low-quality sequences. Conservative thresholds were applied in all cases to increase the reliability of the resulting mutation calls, at the potential expense of excluding some real mutations. Pre-processing was carried out using the Repertoire Sequencing Toolkit (pRESTO) (http://clip.med.yale.edu/pRESTO, manuscript in preparation), and involved:

- Quality control
  1. Removal of low-quality reads (mean Phred quality score <20).
  2. Removal of reads where the primer could not be identified or had a poor alignment score (mismatch rate greater than 0.1).
  3. For the MiSeq data, sets of sequences with identical molecular IDs (corresponding to the same mRNA molecule) were identified. Sets were collapsed into one consensus sequence per set, after discarding those having a mean mismatch rate across all positions >0.2.
  4. For the MiSeq data, the two paired-end reads were assembled into a complete Ig sequence.
  5. Removal of sequences that do not appear in a single sample at least twice.
- Assignment of germline V(D)J segments for each of the Ig sequences: initial V(D)J assignments for each sequence were obtained using IMGT/HighV-QUEST (27). Using these assignments, non-mutated sequences were identified and a V segment germline repertoire for each individual was determined as the set of: (1) V genes that composed at least 0.1% of the sequences, and (2) V gene alleles that composed at least 10% of the assignments to that V gene. Ig sequences that were initially assigned V segments not included in this germline repertoire were then re-assigned to the closest present V segment based on the Hamming distance.
- Removal of non-functional sequences due to the occurrence of a stop codon or/and a reading frame shift between the V gene and the J gene.
- Removal of sequences with more than 30 mutations and masking (replacement with Ns) of positions with Phred quality scores <20.
- Removal of mutations in codons that had more than one mutation, as it is usually not possible to infer the order in which the mutations occurred (and thus the micro-sequence context of the mutations is unknown).
- Identification of clonally related sequences: a two-step approach was applied to identify sequences that were part of a B cell clone (i.e., related through descent from a common ancestor). First, the sequences were divided into groups based on equivalence of their V-gene assignment, J-gene assignment, and the number of nucleotides in their junction. Second, clones were defined within each of these groups as the collection of sequences with junction regions that differed from one sequence to any of the others by no more than three point mutations. The threshold of three was determined after manual inspection of the mutation patterns in resulting clones identified through building lineage trees.

## 4 Discussion

We have constructed new SHM targeting and substitution models using a collection of more than 800,000 synonymous mutations from next-generation Ig sequencing studies. The exclusive use of synonymous mutations allowed us to include mutations from functional Ig sequences without the biasing influence of selection. The large size of the resulting mutation data set allowed us to model targeting and substitution dependencies on the mutating base as well as on two bases upstream and downstream of the mutation. The resulting “S5F” models validate, and also help refine, previously defined SHM hot and cold spots. Figure 4 shows how the classic WRCY/RGYW hot-spot excludes some highly mutable WRCA/TGYW motifs, implying that, as proposed by Rogozin and Diaz (15), WRCH/DGYW could be a better predictor of mutation. However, while the most mutable WRCA/TGYW motifs are even hotter than WRCY/RGYW, others are comparable to neutral motifs. This high variance demonstrates the importance of including higher order dependencies, as we have done.

It has been suggested that nucleotide substitution profiles are also dependent on the micro-sequence context of the mutating base (8, 17). We confirm that the substitution profiles at all nucleotides are highly dependent on neighboring bases and these dependencies are conserved across individuals. Interestingly, the fact that substitution rates depend on surrounding bases may resemble the situation in meiotic mutations as was suggested in the past (9). The ability of the S5F models to estimate mutability and substitution at each of the 1024 DNA 5-mer motifs will allow for detailed, quantitative comparison of SHM with other mutation processes.

A potential source of error in the approach taken here is the existence of novel polymorphisms among the seven individuals studied (Table 1). Since mutation detection depends on comparison with known V and J segments that are part of the IMGT repertoire, undetected polymorphisms will look like mutations. However, any effect on the S5F model is expected to be small relative to the estimated confidence intervals. Based on a new statistical tool to detect novel germline alleles from high-throughput sequencing data (manuscript in preparation), the magnitude of this effect was estimated to be less than ∼1% of the sequences and less than ∼0.1% of the mutations used for the current analysis. The S5F mutability and substitution models presented here were developed using human heavy chain data, and thus may not be valid for light chains or mouse sequences. Given the large amount of sequencing data becoming available, it may be possible to extend the proposed approach to model 7-mers instead of 5-mers. However, even with 5-mers, the values for some motifs had to be inferred because of the limited diversity in germline repertoires. It will be important to estimate the quality of these inferences experimentally. Future experiments might be designed to enrich for non-productively rearranged Ig sequences which could then be sequenced using high-throughput technologies. Since mutations in these sequences are (presumably) not subject to selection, they provide a way to independently estimate substitution profiles and mutabilities for at least some of the motifs inferred in the S5F model. It will be important to confirm that the mutation process operating on these non-productive sequences is equivalent to the process at the productive alleles. This uncertainty is one reason why only productively rearranged Ig sequences were included in the current model.

The targeting and substitution models developed here provide a quantitative description of SHM in the absence of selection, and thus provide an important background for statistical analysis of SHM patterns in experimental data. For example, such models play an important role in quantifying antigen-driven selection in Ig sequences (11, 20), and we have now made the S5F model available as an option on our website for quantifying selection (http://clip.med.yale.edu/baseline). When combined with high-throughput sequencing, it should now be possible to quantify selection for each position of the Ig sequence independently and link these values back to the physical structure of the protein. Following the approach of Brard and Guguen (28), these models could also be incorporated into methods for building lineage trees of B cell clones (29), thus helping to provide insight into the underlying population dynamics of adaptive immunity. The model and method source code are made available at http://clip.med.yale.edu/SHM.

## Conflict of Interest Statement

The authors declare that the research was conducted in the absence of any commercial or financial relationships that could be construed as a potential conflict of interest.

## Supplementary Material

The Supplementary Material for this article can be found online at http://www.frontiersin.org/journal/10.3389/fimmu.2013.00358/abstract
