# Supplementary material for: Models of Somatic Hypermutation Targeting and Substitution Based on Synonymous Mutations from High-Throughput Immunoglobulin Sequencing Data
Source: Front Immunol. 2013 Nov 15;4:358. doi: 10.3389/fimmu.2013.00358 (PMC3828525; doi:10.3389/fimmu.2013.00358)
Supplement: Supplementary file 1 [file 64394_Kleinstein_Presentation1.PDF]

Supporting Information for: "Models of somatic hypermutation targeting and substitution based on synonymous mutations from high-throughput Immunoglobulin sequencing data."

Gur Yaari[1,2], Jason Vander Heiden[3], Mohamed Uduman[2], Daniel Gadala-Maria[3], Namita Gupta[3], Joel N.H. Stern[4,5], Kevin C. O'Connor[4,6], David A. Hafler[4,7], Uri Laserson[8], Francois Vigneault[9], and Steven H. Kleinstein*[2,3]

[1]Bioengineering program, Faculty of engineering, Bar Ilan University, Ramat Gan, Israel
[2]Department of Pathology, Yale School of Medicine, New Haven, CT, USA
[3]Interdepartmental Program in Computational Biology and Bioinformatics, Yale University, New Haven, CT, USA
[4]Department of Neurology, Yale School of Medicine, New Haven, CT, USA
[5]Department of Science Education, Hofstra Northshore-LIJ School of Medicine, Hempstead, NY, USA
[6]Human and Translational Immunology Program, Yale School of Medicine, New Haven, CT, USA
[7]Department of Immunobiology, Yale School of Medicine, New Haven, CT, USA
[8]Department of Genetics, Harvard Medical School, Boston, MA, USA
[9]AbVitro, Inc., Boston, MA, USA

October 25, 2013

*to whom correspondence should be addressed. Tel: +1 (203) 785-6685; Email: steven.kleinstein@yale.edu

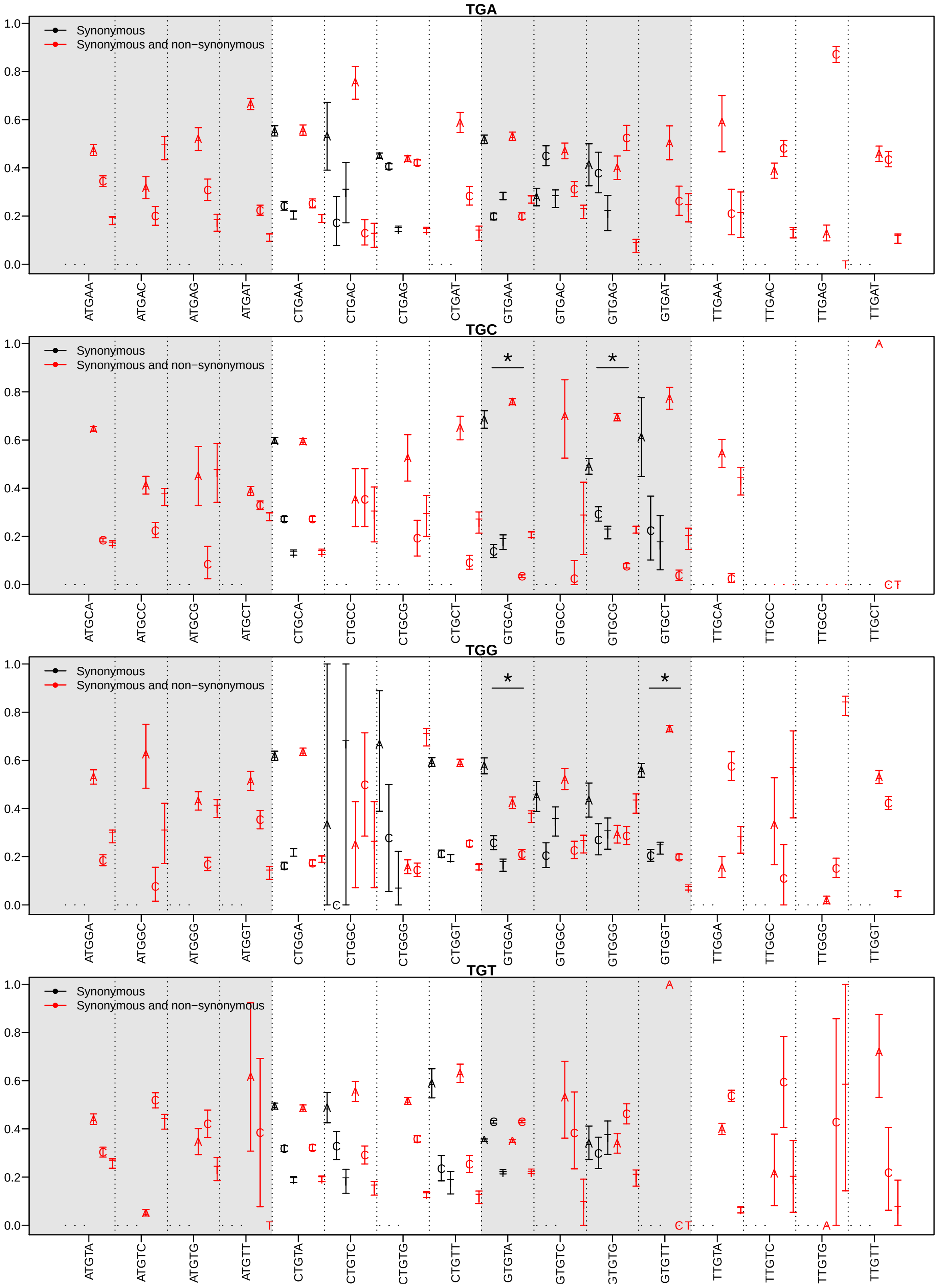

Figure S1: **Including replacement mutations alters the estimated substitution model.** Nucleotide substitution models were constructed using silent mutations only (S5F, black symbols) or using both replacement and silent mutations (RS5F, red symbols). Each plot shows a subset of the model for G when preceded by T. Profiles are shown as a function of the immediately downstream nucleotide (4 individual plots) and the 16 possible base combinations two bases upstream or downstream (columns within each plot). Only substitution rates that could be estimated directly from the data are shown (i.e., inferred values are not included). Bars indicate the 95% confident interval for the measured value.

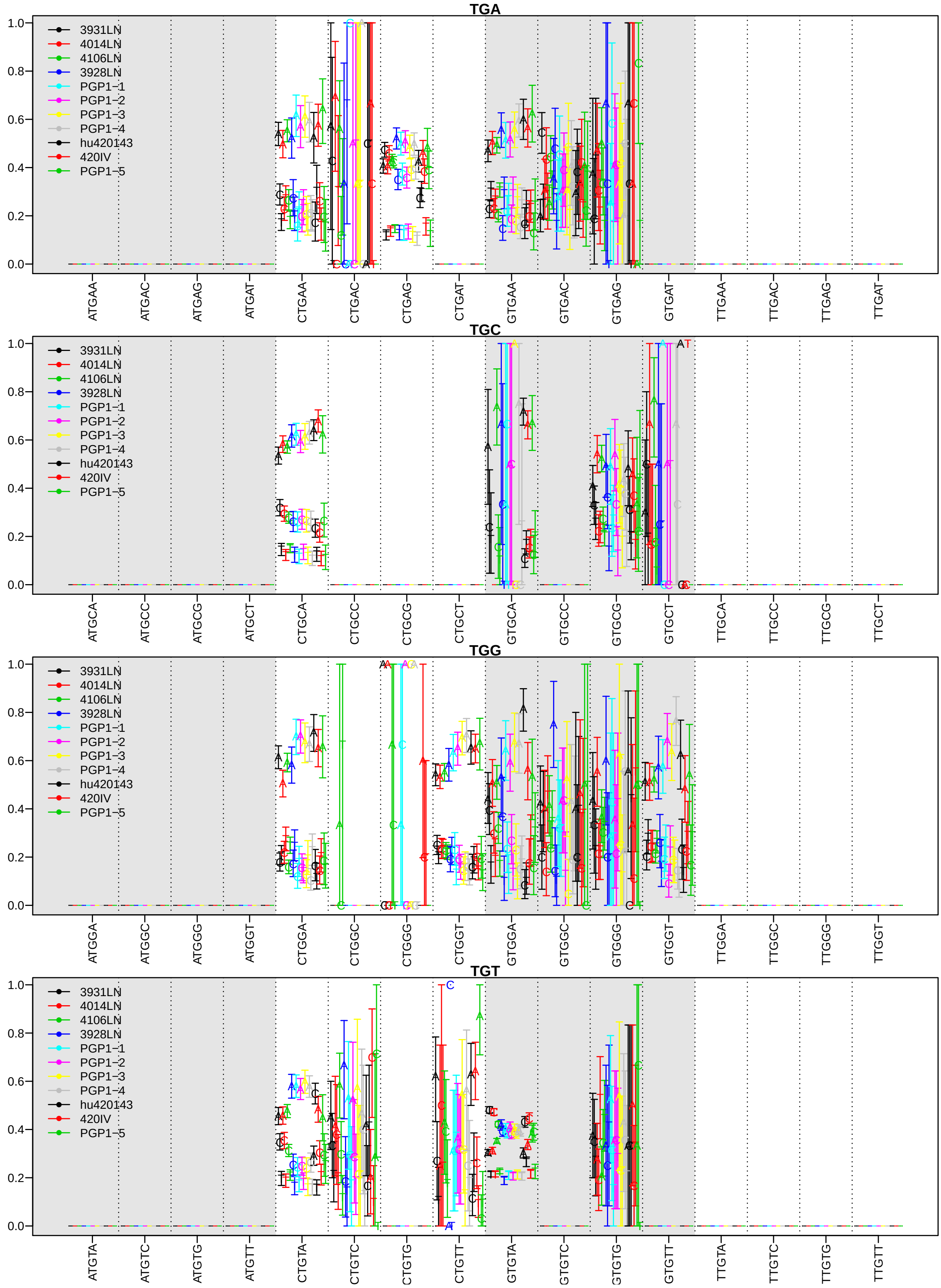

Figure S2: **The substitution model is highly reproducible across different individuals** . Nucleotide substitution models were constructed separately for each sample in Table 1 (different colors). Each plot shows a subset of the model for G when preceded by T. Profiles are shown as a function of the immediately downstream nucleotide (4 individual plots) and the 16 possible base combinations two bases upstream or downstream (columns within each plot). Only substitution rates that could be estimated directly from the data are shown (i.e., inferred values are not included). Bars indicate the 95% confident interval for the measured value.

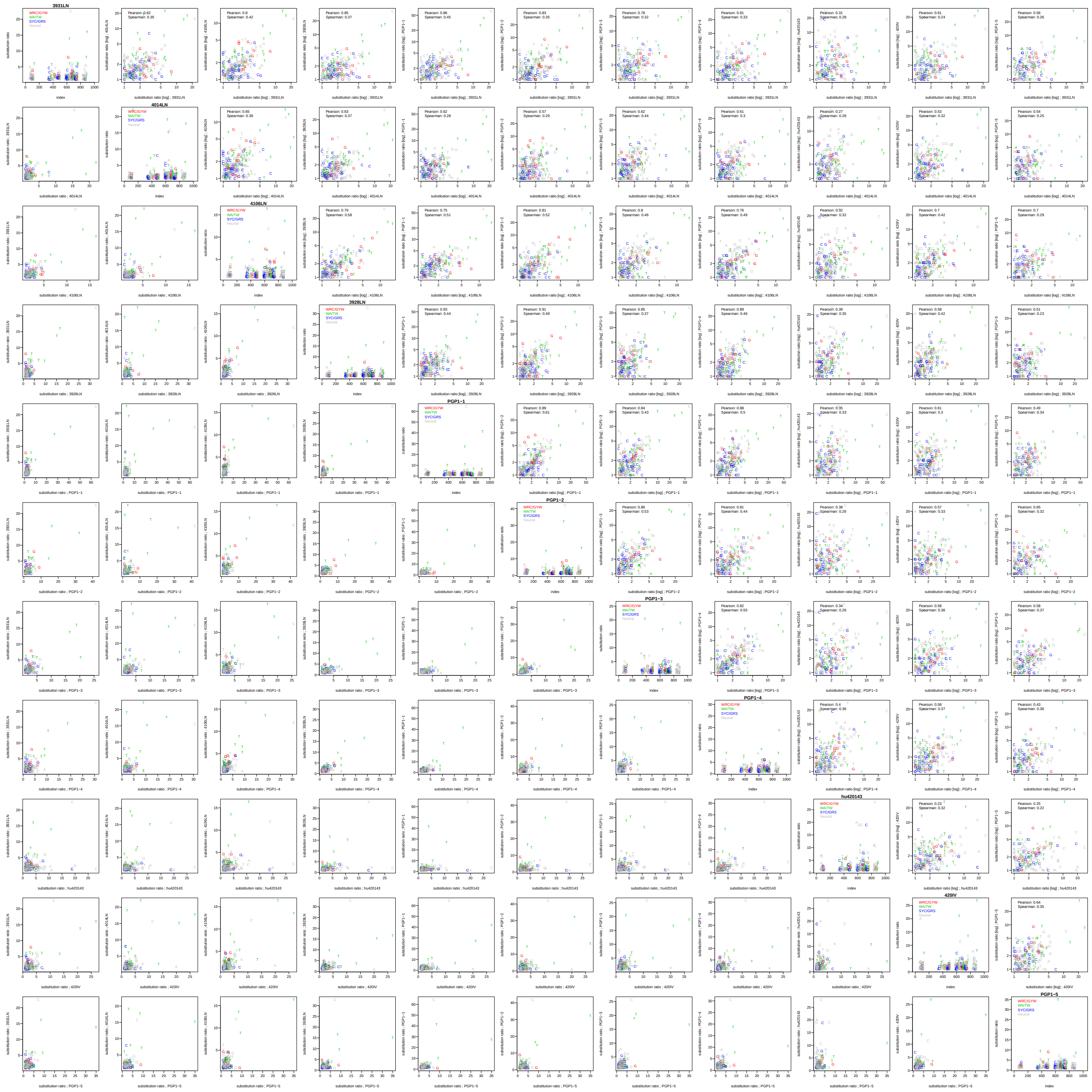

Figure S3: **The S5F substitution model is highly reproducible across different individuals** Substitution models were constructed independently for each of the samples listed in Table 1 (main text). Transition bias scores (maximum substitution rate divided by the second highest substitution rate) for all 5-mer motifs are shown along the diagonal. Scores are ranked (from lowest to highest) and color coded by their category (WRC/GYW are red, SYC/GRS are blue, WA/TW are green and the rest are gray). Symbols indicate the mutated nucleotide (in the center of the 5-mer). Correlations between the scores for all 5-mer motifs across individuals are shown in the upper (log-log scale) and lower (linear scale) triangles.

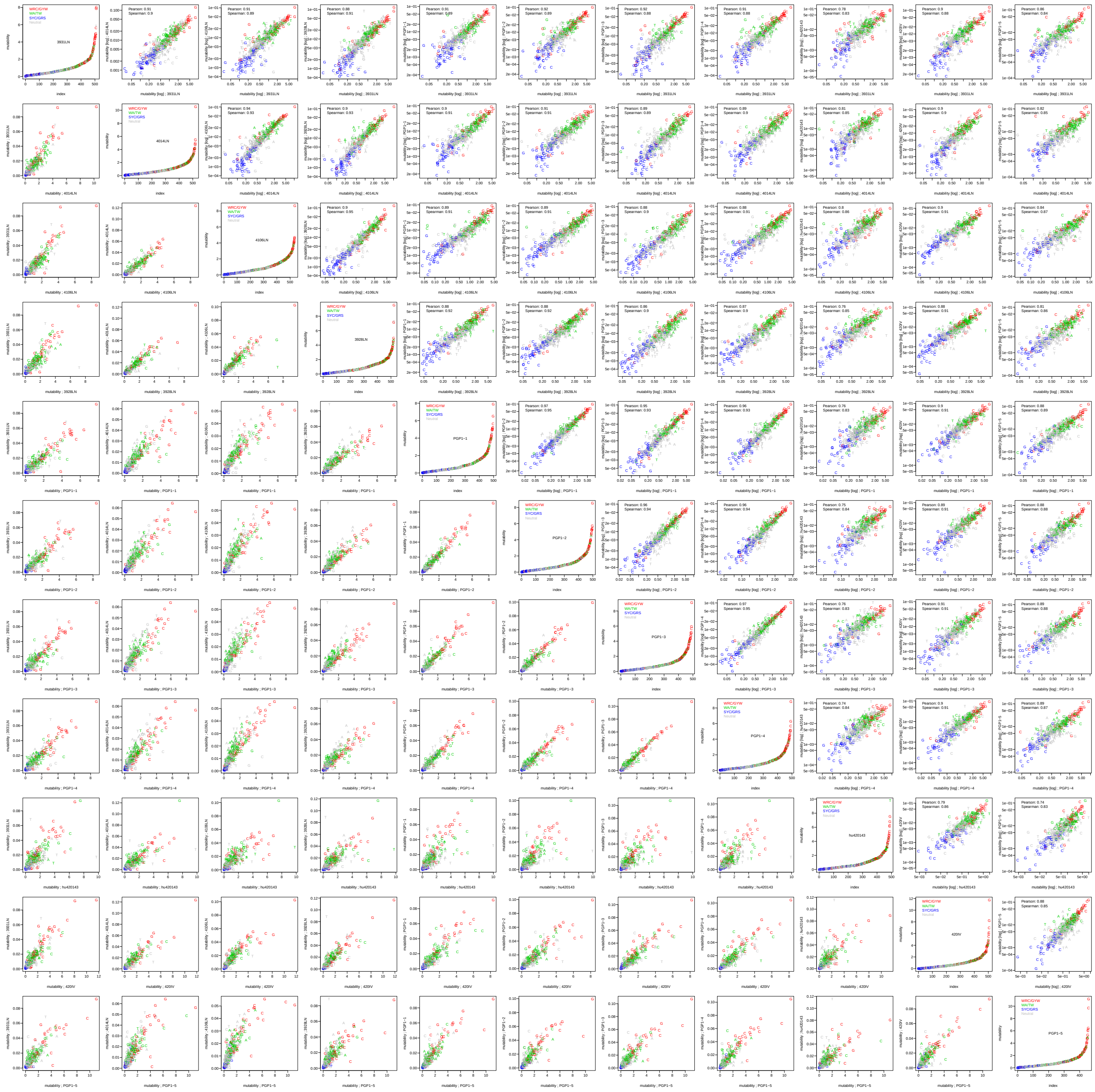

Figure S4: **The S5F targeting model is consistent across individuals.** Targeting models were constructed independently for each of the samples listed in Table 1 (main text). Estimated values for all 5-mer motifs are shown along the diagonal. Mutability values are ranked (from lowest to highest) and color coded by their category (WR<u>C</u>/<u>G</u>YW are red, SY<u>C</u>/<u>G</u>RS are blue, W<u>A</u>/<u>T</u>W are green and the rest are gray). Symbols indicate the mutated nucleotide (in the center of the 5-mer). Correlations between the mutabilities for all 5-mer motifs across individuals are shown in the upper (log-log scale) and lower (linear scale) triangles.

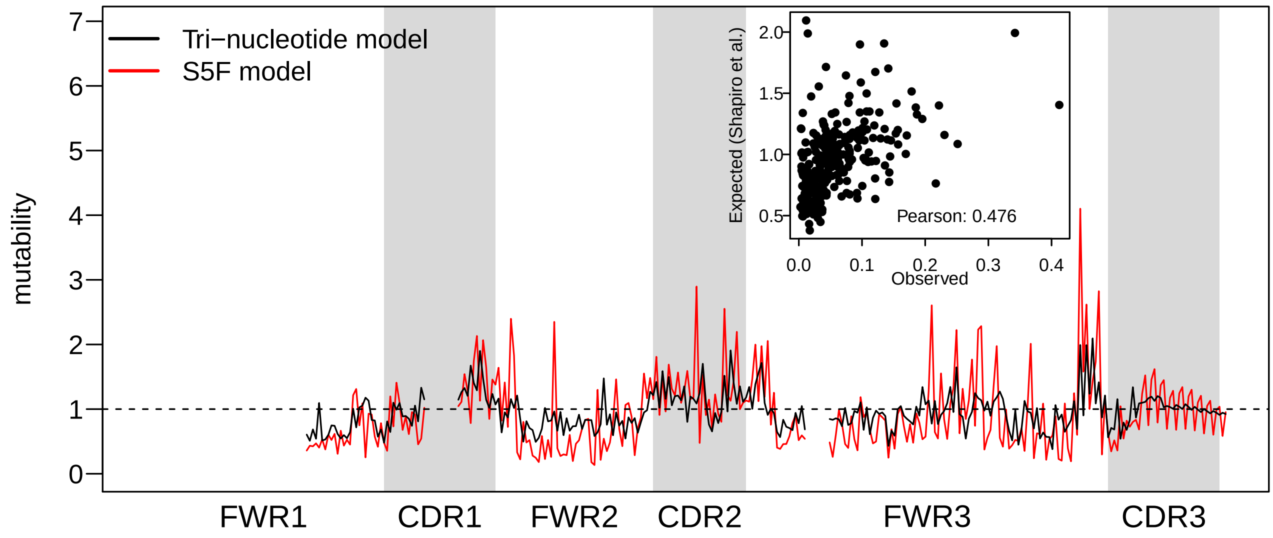

Figure S5: **Comparison between expected and observed somatic hypermutation targeting for the tri-nucleotide model**

The predicted mutability from the tri-nucleotide model of Shapiro et al. 2003 (black line) and the S5F model (red line) for each position in the Ig sequence (IMGT-aligned along the x-axis).Expectations are based on the observed repertoire of one sample (3931LN). The correlation between the tri-nucleotide predictions and the observed mutation frequency across positions (points) is shown in the inset (compare with subplot of main figure Figure 5B).
